# Optimization of Emulsification Parameters for Preparing Hydrogel Beads Based on an Enzymatically Cross-Linkable Poly(aspartamide) Derivative

**DOI:** 10.3390/gels12030230

**Published:** 2026-03-11

**Authors:** Danqing Liu, Guangyan Zhang

**Affiliations:** 1 School of Materials and Chemical Engineering, Hubei University of Technology, Wuhan 430068, China; liudanqinghbut@163.com; 2 Hubei Provincial Key Laboratory of Green Materials for Light Industry, Hubei University of Technology, Wuhan 430068, China

**Keywords:** hydrogel beads, emulsification, enzyme cross-linking, poly(aspartamide) derivative, in situ hydrogel

## Abstract

In this study, phenolic hydroxyl-functionalized poly(α,β-[*N*-(2-hydroxyethyl)-D, L-aspartamide]) (PHEA-HP) was used to prepare hydrogel beads via an emulsion-enzymatic gelation process. The effects of the preparation conditions on the size and size distribution span of the hydrogel beads were investigated. Initially, single-factor experiments were conducted to determine the range of preparation conditions for hydrogel beads. Subsequently, a Box–Behnken design combined with response surface methodology (BBD–RSM) was employed to optimize the emulsification parameters for preparing hydrogel beads, with three numerical independent variables (oil-to-water ratio, homogenization rate, and Span 80 dosage) and two responses (size and size distribution span). The results indicated that the size distribution span fit the quadratic model well and was more sensitive to the three independent variables than size. The optimal preparation conditions were validated to be an oil-to-water ratio of 10.3, a homogenization rate of 2930 rpm, and a Span 80 dosage of 2.0%. At the optimum point, the prepared PHEA-HP hydrogel beads were spherical, with an average size of 14.0 ± 0.2 μm and a size distribution span of 0.185 ± 0.010.

## 1. Introduction

In recent years, hydrogel beads have been extensively studied and have demonstrated broad potential across various fields, including the food industry [1,2], biomedicine [3,4], environmental protection [5,6], catalyst development [7], and separation technology [8]. Depending on specific application requirements, a wide range of polymers can be used to prepare hydrogel beads. Generally, for applications closely related to human health, such as food and biomedicine, alginate [9], chitosan [10], and gelatin [11,12] are commonly used due to their biodegradability and non-toxicity. In contrast, for applications requiring hydrogel beads with excellent stability, polymers with carbon–carbon backbones, such as polyvinyl alcohol [13] and polyacrylamide [14], are often preferred.

Cross-linking is a crucial process for transforming polymer aqueous solutions into hydrogels. To prepare spherical hydrogel beads, polymer aqueous solutions typically form small droplets, which then undergo the solution-gel transition via in situ cross-linking. In situ cross-linking can be triggered by ions [15], temperature [16], photo excitation (e.g., visible light) [17], or enzymes [18], and is generally classified into ionic and covalent cross-linking. A typical example of ionic cross-linking is the in situ gelation of sodium alginate aqueous solution with Ca^2+^, which not only spontaneously forms spherical gels in the aqueous phase [19] but also completes gelation rapidly, often in just a few seconds [20]. Representative examples of covalent cross-linking include the self-cross-linking of thiol groups (-SH) to form disulfide bonds (-S-S-) under aerobic conditions [21], as well as enzymatic cross-linking [22,23,24].

Enzymes commonly used for in situ cross-linking include tyrosinase, transglutaminase, and horseradish peroxidase (HRP). Transglutaminase can induce in situ cross-linking of gelatin [25,26], while horseradish peroxidase (HRP) can typically combine with hydrogen peroxide (H_2_O_2_) to promote in situ cross-linking of polymers containing phenolic moieties [27,28]. In the HRP/H_2_O_2_ in situ cross-linking system, it is worth noting that the gelation time can be flexibly adjusted over a wide range by regulating the concentrations of HRP or H_2_O_2_ [29]. This flexible and tunable gelation time facilitates the preparation of hydrogel beads.

Hydrogel beads can be prepared using various methods, including dropwise dispensing [30,31], coaxial needle techniques [32], emulsification [33], and microfluidics [34,35]. For dropwise dispensing and coaxial needle methods, millimeter-sized hydrogel beads can often be obtained [36]. For emulsification and microfluidics methods, micrometer-sized or nanometer-sized hydrogel beads can usually be prepared. For example, Wada’s group prepared a water-in-oil emulsion by adding a regenerated cellulose aqueous solution to decane (oil phase) at 120 °C, using Span 80 or Span 40 as an emulsifier. Gelation was completed by cooling, resulting in cellulose hydrogel beads with an average diameter of 9.4–21.2 μm [37]. Sivan et al. employed a simple one-step emulsification method with mineral oil as the oil phase to prepare small alginate beads (127–257 µm) via internal gelation [38].

**Scheme 1:**
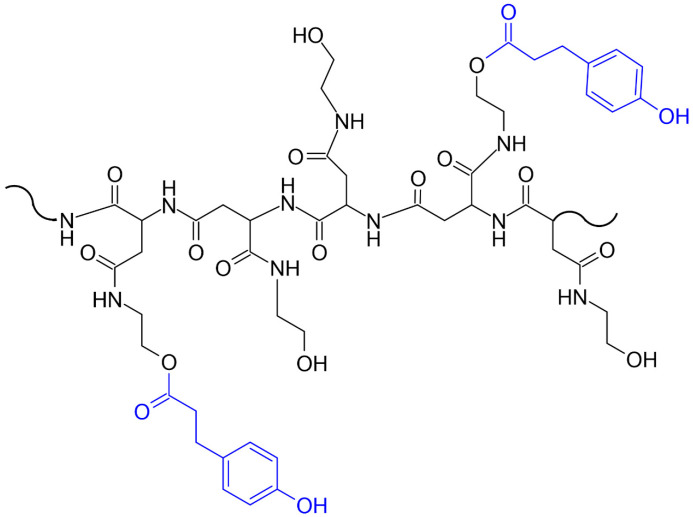
Chemical structure of PHEA-HP. The HP moieties are highlighted in blue.

In our previous work, we successfully synthesized an enzymatically cross-linkable poly(aspartamide) derivative, PHEA-HP (Scheme 1), which exhibited excellent cytocompatibility and blood compatibility. The gelation time of PHEA-HP could be easily controlled by adjusting the concentrations of HRP or H_2_O_2_. Furthermore, hydrogel beads with uniform spherical shapes and diameters ranging from 1.9 mm to 2.9 mm were fabricated using a PHEA-HP/HRP/H_2_O_2_ formulation (gelation time: approximately 50 s) and a coaxial needle [39]. However, the millimeter-scale size of these PHEA-HP hydrogel beads limits their applications in certain fields.

In recent years, there have been reports on the preparation of micrometer-sized hydrogel beads via an emulsion-enzymatic gelation process using various polymers, such as gelatin [40], hyaluronic acid [40], and pea protein [41]. However, the effects of emulsification parameters on the size and size distribution span have not been systematically investigated. Furthermore, no studies have reported the use of poly(aspartamide) derivatives for this purpose. In this study, the enzymatically cross-linkable poly(aspartamide) derivative PHEA-HP was employed for the first time to prepare micrometer-sized hydrogel beads via an emulsion-enzymatic gelation process. The effects of emulsification parameters on the size and size distribution span of the prepared hydrogel beads were also investigated. Initially, single-factor experiments were conducted to preliminarily determine the range of preparation conditions for the hydrogel beads. Subsequently, the preparation conditions of PHEA-HP hydrogel beads were optimized using a Box–Behnken design combined with response surface methodology (BBD-RSM), with three numerical independent variables (oil-to-water ratio, homogenization rate, and Span 80 dosage). Finally, the optimal conditions were validated through particle size analysis and scanning electron microscopy (SEM).

## 2. Results and Discussion

### 2.1. Synthesis and Characterization of PHEA-HP

Enzymatically cross-linkable poly(aspartamide) derivative PHEA-HP was synthesized by the esterification of 3-(4-hydroxyphenyl)propionic acid (HP) with poly(α,β-[N-(2-hydroxyethyl)-D, L-aspartamide]) (PHEA). The ^1^H NMR of PHEA-HP (Figure S1) was consistent with that reported in our previous work [39], confirming the chemical structure of PHEA-HP. As shown in Figure S1, peak b′ (4.0 ppm) indicates the formation of ester linkages between PHEA and HP. Based on the integral ratio of peak at 6.6 ppm (peak i, 2H in HP moieties) to peak at 3.1 ppm (peak a and a′, 2H in PHEA), the molar percentage of introduced HP moieties in PHEA-HP was calculated to be 7.6%. Using gel permeation chromatography (GPC) calibrated with polystyrene standards, the number-average molecular weight (M_n_) and the polydispersity index (PDI) of the obtained PHEA-HP were determined to be 89.1 kDa and 1.22, respectively. Although PHEA-HP contains benzene rings in its side chains, its hydrophilicity and side chain length are different from those of polystyrene. This means that, with the same backbone length, the elution volume of PHEA-HP may differ from that of polystyrene, resulting in a certain difference between the relative and absolute molecular weights of PHEA-HP. Gelation time of PHEA-HP/HRP/H_2_O_2_ aqueous solution (PHEA-HP concentration: 6.0 wt.%) as a function of the concentration of HRP and H_2_O_2_ is shown in Figure S2.

### 2.2. Single-Factor Experiments

The purpose of the single-factor experiments was to screen out the range of preparation conditions that could form PHEA-HP hydrogel beads. The appearance of the hydrogel beads prepared under different conditions was evaluated using an optical microscope. Considering that the freshly prepared PHEA-HP/HRP/H_2_O_2_ aqueous solution requires a 3 min homogenization period to form a w/o emulsion upon addition to liquid paraffin containing Span 80, the gelation time of the selected formulation should be no less than 3 min. The gelation time, determined by the vial-tilting method, is defined as the time at which the PHEA-HP/HRP/H_2_O_2_ aqueous solution no longer flows within 20 s after the vial is inverted. Notably, in situ cross-linking begins before the recorded gelation time, accompanied by an increase in viscosity, while the solution remains fluid. It is well known that as the viscosity of the dispersed phase increases, droplet disruption and breakup become more difficult, thereby increasing the risk of re-coalescence. The re-coalescence of droplets often leads to larger or irregular hydrogel beads. In addition, since the addition of PHEA-HP/HRP/H_2_O_2_ aqueous solution and the operation of the homogenizer also require time in practical operation, a formulation (PHEA-HP: 6.0 wt.%, HRP: 0.5 units/mL, H_2_O_2_: 10 mM) with a gelation time of about 6 min was chosen to prepare hydrogel beads for subsequent investigation. The gel yield of this formulation was ~67%, and the swelling kinetics showed an equilibrium swelling ratio of approximately 39.1 (Figure S3). Gel yield is primarily limited by incomplete cross-linking. Typically, as the gelation time of the formulation increases, the degree of cross-linking decreases, leading to a high swelling ratio and an increase in uncross-linked PHEA-HP. The uncross-linked PHEA-HP will be lost during the water-soaking process.

In two-phase liquid–liquid systems, the cohesive stress and disruptive stress compete with each other during droplet formation. Cohesive stress is proportional to the interfacial tension but inversely proportional to size. Disruptive stress depends on the external flow conditions. For a spherical droplet, it is stable when cohesive stress is greater than disruptive stress. When disruptive stress exceeds cohesive stress, droplet breakup occurs. For preparing w/o emulsions, oil serves as the dispersion medium, and the oil-to-water ratio primarily affects droplet coalescence. In this work, the cohesive stress is associated with the emulsifier Span 80, while the disruptive stress is associated with the homogenization rate. Therefore, the oil-to-water ratio, homogenization rate, and Span 80 dosage are crucial to the preparation of PHEA-HP hydrogel beads.

#### 2.2.1. Effect of Oil-to-Water Ratio

The effect of the oil-to-water ratio on the formation of PHEA-HP hydrogel beads was investigated at a range of 4 to 12 (*v*/*v*, paraffin liquid/aqueous solution) with a homogenization rate of 3000 rpm and 2% Span 80 (*w*/*v* in paraffin liquid).

**Figure 1:**
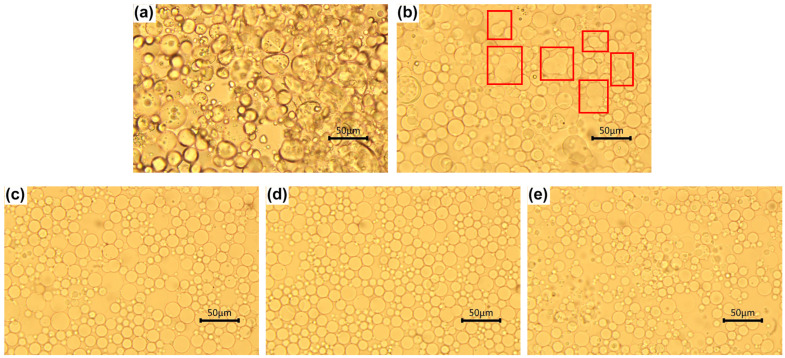
Optical images of PHEA-HP hydrogel beads prepared at varying oil-to-water ratios: (**a**) 4, (**b**) 6, (**c**) 8, (**d**) 10, and (**e**) 12 (homogenization rate: 3000 rpm; Span 80: 2% *w*/*v* in paraffin liquid). Irregular hydrogels are highlighted with the red rectangles.

As shown in Figure 1, at an oil-to-water ratio of 4, irregular hydrogels formed. This may be due to the high dispersed-phase content, which makes it difficult to disperse the PHEA-HP/HRP/H_2_O_2_ aqueous solution in paraffin (Figure 1a). When the oil-to-water ratio was increased to 6, hydrogel beads with a diameter of 14.9 ± 5.5 μm were observed, accompanied by some irregular hydrogel beads (Figure 1b). When the oil-to-water ratio was further increased to the range of 8 to 12 (Figure 1c–e), hydrogel beads could be formed well. The prepared hydrogel beads in Figure 1c, Figure 1d and Figure 1e have diameters of 13.9 ± 3.9 μm, 13.4 ± 3.3 μm, and 13.0 ± 3.1 μm, respectively. Figure S4 showed that the size distribution was more uniform in the range of 8–12. Therefore, in subsequent BBD-RSM experiments, the oil-to-water ratio was used as the independent variable A, ranging from 8 to 12.

#### 2.2.2. Effect of Homogenization Rate

The effect of homogenization rate on the formation of PHEA-HP hydrogel beads was investigated over a range of 1500–3500 rpm, with an oil-to-water ratio of 10 and 2% Span 80 (*w*/*v* in paraffin liquid).

**Figure 2:**
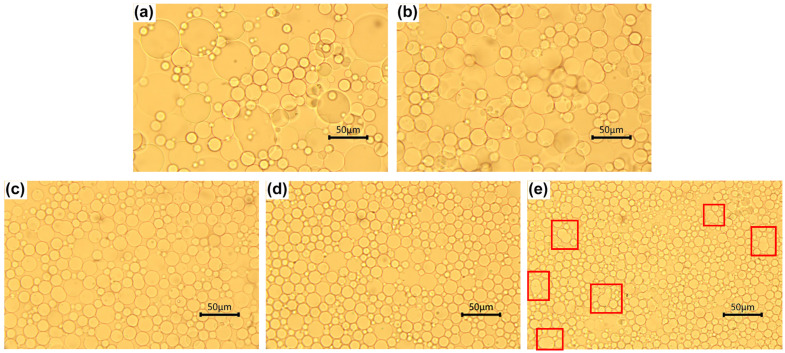
Optical images of PHEA-HP hydrogel beads prepared at varying homogenization rates: (**a**) 1500 rpm, (**b**) 2000 rpm, (**c**) 2500 rpm, (**d**) 3000 rpm, and (**e**) 3500 rpm (oil-to-water ratio: 10; Span 80: 2% *w*/*v* in paraffin liquid). Irregular hydrogels are highlighted with the red rectangles.

As shown in Figure 2, hydrogel beads formed at all homogenization rates tested, but those at low rates often had large diameters. The diameter of hydrogel beads in Figure 2b (2000 rpm) was 18.1 ± 4.8 μm, and the regularity of the spheres is not as good as in Figure 2c,d. The prepared hydrogel beads in Figure 2c (2500 rpm) and 2d (3000 rpm) had diameters of 13.9 ± 3.8 μm and 12.9 ± 3.1 μm, respectively. The representative size distributions corresponding to Figure 2b–d are shown in Figure S5. At a homogenization rate of 3500 rpm (Figure 2e), the diameter was 11.7 ± 1.7 μm, although the proportion of irregular hydrogel beads (in red rectangles) increased.

Overall, as the homogenization rate increased, the obtained hydrogel beads became smaller. Therefore, in subsequent BBD-RSM experiments, the homogenization rate was used as the independent variable B, ranging from 2500 rpm to 3500 rpm.

#### 2.2.3. Effect of Span 80 Dosage

The effect of Span 80 dosage on the formation of PHEA-HP hydrogel beads was investigated over the range of 1–5% (*w*/*v* in paraffin liquid), with an oil-to-water ratio of 10 and a homogenization rate of 3000 rpm.

**Figure 3:**
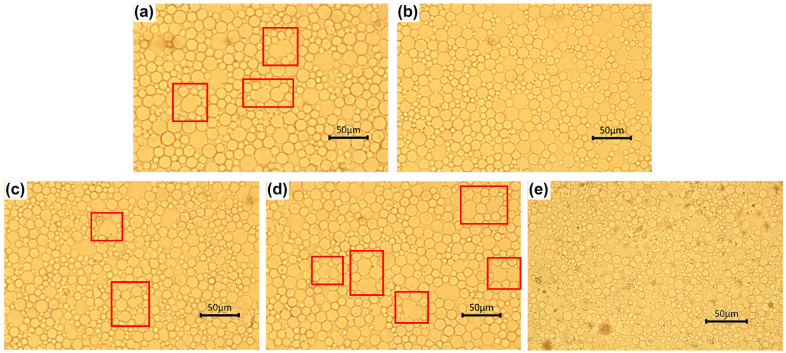
Optical images of PHEA-HP hydrogel beads prepared at varying Span 80 dosages (*w*/*v* in paraffin liquid): (**a**) 1%, (**b**) 2%, (**c**) 3%, (**d**) 4% and (**e**) 5% (oil-to-water ratio: 10; homogenization rate: 3000 rpm). Irregular hydrogels are highlighted with the red rectangles.

As seen in Figure 3a, at a Span 80 dosage of 1%, although a small number of droplets fused during gelation, resulting in poor sphericity in some hydrogels (highlighted by red rectangles), the overall formation of hydrogel beads was good, with a diameter of 13.4 ± 2.9 μm. When the Span 80 dosage was increased to 2%, small hydrogel beads (12.9 ± 3.0 μm) can also be obtained, and irregular hydrogels are significantly reduced. When the Span 80 dosage was further increased to 3% (Figure 3c) and 4% (Figure 3d), the average diameter of the hydrogel beads did not decrease significantly, remaining around 12 μm, but irregular hydrogels reappeared and increased. The representative size distributions corresponding to Figure 3a,b are shown in Figure S6.

Interestingly, when the Span 80 dosage reached 5%, the resulting hydrogel beads, although smaller in size, contained many broken and irregular hydrogels. This may be due to excessive Span 80 accumulating at the oil-water interface, making it difficult to clean. Therefore, in subsequent BBD-RSM experiments, the Span 80 dosage was used as the independent variable C, ranging from 1% to 3%.

### 2.3. Optimization of the Preparation Conditions Based on BBD–RSM

Based on the results of the single-factor experiments above, the preparation conditions of PHEA-HP hydrogels were optimized using the BBD-RSM. The results “D[3,2]” (Y1) and “span value” (Y2) obtained from the Mastersizer 2000 particle size analyzer were used as evaluation indicators to evaluate the size (diameter) and size distribution span of the hydrogel beads, respectively (Table 1).

**Figure 4:**
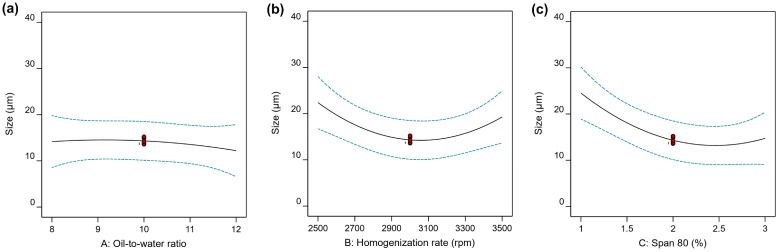
Influences of (**a**) oil-to-water ratio, (**b**) homogenization rate, and (**c**) Span 80 dosage on the size (Y1) of PHEA-HP hydrogel beads.

**Figure 5:**
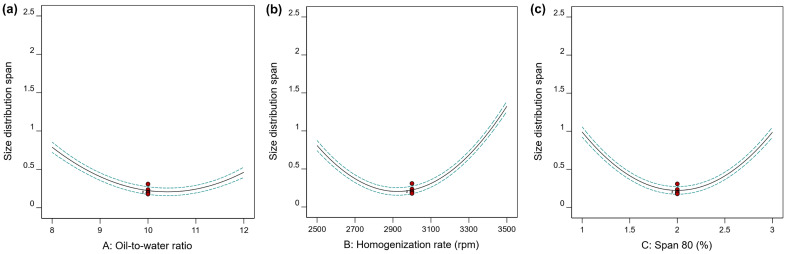
Influences of (**a**) oil-to-water ratio, (**b**) homogenization rate, and (**c**) Span 80 dosage on the size distribution span (Y2) of PHEA-HP hydrogel beads.

**Figure 6:**
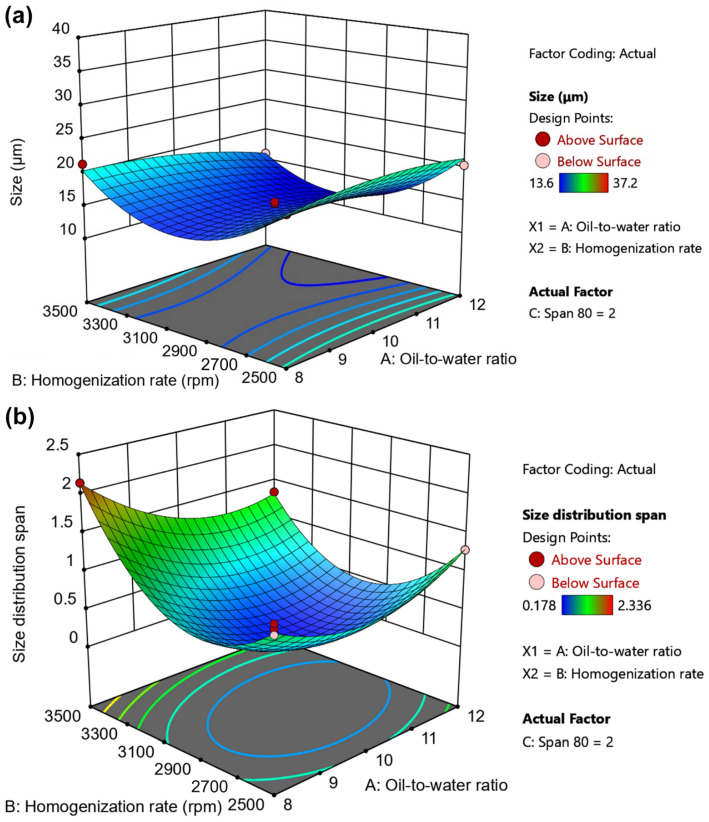
Effects of oil-to-water ratio and homogenization rate on (**a**) size and (**b**) size distribution span.

**Figure 7:**
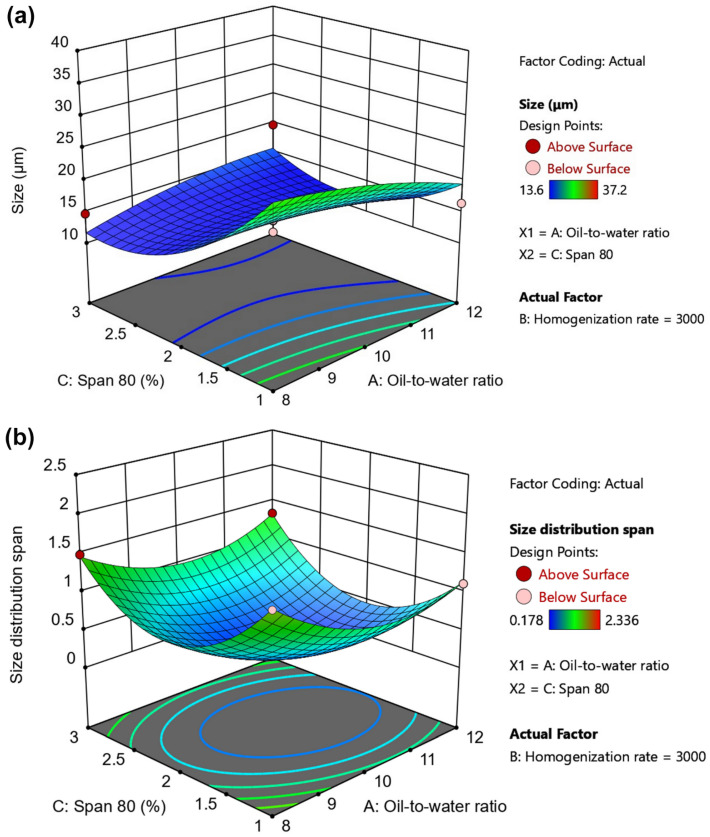
Effects of oil-to-water ratio and Span 80 dosage on (**a**) size and (**b**) size distribution span.

**Figure 8:**
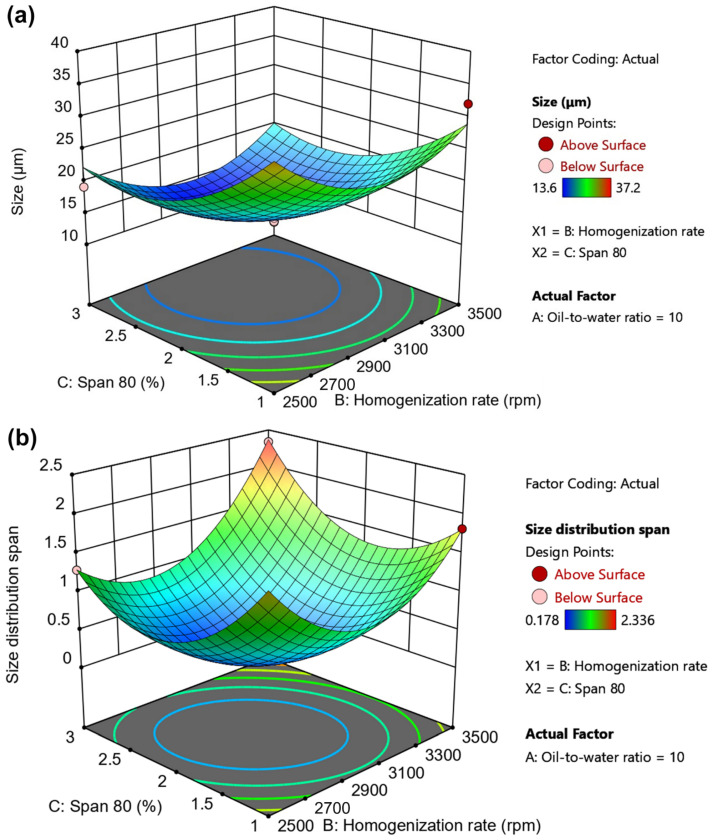
Effects of homogenization rate and Span 80 dosage on (**a**) size and (**b**) size distribution span.

Seventeen randomized experimental runs were generated by the Box–Behnken design, and the results Y1 (D[3,2]) and Y2 (span value) obtained from all experiments are summarized in Table 2. The size (Y1) of PHEA-HP hydrogel beads varied from 13.6 μm (Run 2) to 37.2 μm (Run 10), while size distribution changed between 0.178 (Run 5) and 2.336 (Run 4). The representative size distributions are shown in Figure S7. The analysis of variance (ANOVA) test is provided in Table 3. The main effects of the independent variables are shown in Figure 4 and Figure 5. 3D response surface plots produced by Design-Expert are shown in Figure 6, Figure 7 and Figure 8.

#### 2.3.1. Model Fitting of RSM

In the analysis of variance (ANOVA) test, Design-Expert suggested quadratic models for both Y1 (size) and Y2 (size distribution span). The following Equations (1) and (2) present the models of Y1 and Y2, respectively, in terms of coded factors:

Y1 = 14.32 − 0.9750A − 1.55B − 4.90C − 1.15AB + 2.95AC + 0.500BC − 1.14 A^2^ + 6.52B^2^ + 5.31C^2^ (1)

Y2 = 0.2236 + 0.163375A + 0.2585B + 0.001625C + 0.24575AB + 0.09AC + 0.27825BC + 0.4022A^2^ + 0.84345B^2^ + 0.7662C^2^ (2)

The model was significant (*p*-value = 0.0389) for size (Y1), but both R^2^ (0.8395) and adjusted R^2^ (0.6331) were not satisfactory. Of the three independent variables, only C (Span 80 dosage, *p*-value = 0.0100) was found to be significant. Furthermore, lack of fit was also significant for Y1. These results indicate that the quadratic model may have a poor fit to the size of PHEA-HP hydrogel beads, which may be ascribed to the wide size distribution of hydrogel beads under certain conditions (Run 4, Run 10, Run 12, and Run 17). A large span value in the size distribution usually indicates poor size uniformity of hydrogel beads, which may lead to a poor match between the actual results and the model.

Interestingly, the model was significant (*p*-value < 0.0001) for the size distribution span (Y2). The non-significant lack of fit value (0.6560), R^2^ (0.9981), and adjusted R^2^ (0.9956) indicate that the quadratic model fits the experimental data well and is suitable for predicting the size distribution span (Y2) of PHEA-HP hydrogel beads. Furthermore, the predicted R^2^ (0.9886) and PRESS (0.0930) confirm that the Y2 model is robust. Among three independent variables, A (oil-to-water ratio) and B (homogenization rate) were found to be significant, except for C (Span 80 dosage, *p*-value = 0.9251). Interestingly, the interactions between A and C, as well as between B and C, were also found to be significant for Y2. In fact, all terms are significant, except C (Span 80 dosage). Moreover, the low coefficient of variation (C.V.) in Y2 (<5%) indicates the reliability of the experimental data.

Therefore, the focus of subsequent discussions and optimization will be on the size distribution span (Y2), while size (Y1) will serve only as a reference for comparison with Y2.

#### 2.3.2. Impacts of Independent Variables on the Size

Although the quadratic model did not fit the experimental data for size (Y1) very well, the influences of three independent variables on the size of PHEA-HP hydrogel beads can still be observed by combining optical microscopy results.

Figure 4a shows the effect of the oil-to-water ratio on Y1 at a homogenization rate of 3000 rpm and a Span 80 dosage of 2%. Its effect on size was mild. Figure 4b shows the effect of homogenization rate on Y1 under an oil-to-water ratio of 10 and a Span 80 dosage of 2%. As the homogenization rate increased, the size first decreased and then increased, reaching its minimum near 3000 rpm. However, as shown in Figure 2, the size gradually decreased as the homogenization rate increased from 2500 rpm to 3500 rpm, although the regularity of the hydrogel obtained at 3500 rpm was not as good as that at 3000 rpm. A similar effect of Span 80 dosage on Y1, as the homogenization rate, was observed at 3000 rpm and an oil-to-water ratio of 10 (Figure 4c).

#### 2.3.3. Impacts of Independent Variables on Size Distribution

The main influences of three independent variables on the size distribution span (Y2) are shown in Figure 5. Figure 5a shows the effect of the oil-to-water ratio on Y2 at a homogenization rate of 3000 rpm and a Span 80 dosage of 2%. As shown in Figure 5a, the effect of the oil-to-water ratio on size distribution span (Y2) is different from its effect on size (Y1). As the oil-to-water ratio increased from 8 to 12, size distribution span first decreased and then increased, while size did not change significantly (Figure 4a).

The other two independent variables, homogenization rate (Figure 5b) and Span 80 dosage (Figure 5c), have effects on the size distribution span similar to those of the oil-to-water ratio, both showing an initial decrease followed by an increase. This may be related to the generation of many tiny, fragmented, or irregular hydrogels under conditions of high homogenization rates (Figure 2e) or high Span 80 dosage (Figure 3e).

#### 2.3.4. 3D Surface Plots of Independent Variables

To understand the combined effects of the independent variables for size and size distribution span, 3D response surface plots were also drawn, as shown in Figure 6, Figure 7 and Figure 8. Figure 6a and Figure 6b shows the interactions between the oil-to-water ratio and homogenization rate on size (Y1) and size distribution span (Y2), respectively. Figure 6a,b indicates that increasing the homogenization rate initially decreases both Y1 and Y2. When the homogenization rate reached around 3000 rpm, both Y1 and Y2 began to increase.

The oil-to-water ratio has a limited effect on size, especially at low homogenization rates (e.g., 2500–2700 rpm), but a significant effect on size distribution span regardless of the homogenization rate. Table 3 also shows that the interaction effect of oil-to-water ratio and homogenization rate on Y2 (size distribution span) is significant (*p*-value < 0.0001). Generally speaking, when the oil-to-water ratio and homogenization rate are both low, due to insufficient space and shear force, the aqueous solution is difficult to disperse effectively into small droplets in liquid paraffin, or the resulting droplets are large and exhibit a wide size distribution. As the oil-to-water ratio increases, droplet dispersion improves, making it easier to form smaller droplets. The increased homogenization rate enhances disruptive stress, further promoting the formation of small droplets with a narrow size distribution (2500–3000 rpm). However, an excessively high homogenization rate provides sufficient shear force to facilitate the formation of smaller droplets, but it also creates a larger surface area. This results in an insufficient amount of emulsifier to stabilize the droplets, leading to droplet coalescence. Notably, the viscosity of PHEA-HP droplets continues to increase due to in situ cross-linking, further exacerbating droplet coalescence. An excessively high oil-to-water ratio not only reduces efficiency but also results in uneven droplet distribution in liquid paraffin, which is also detrimental to obtaining droplets of uniform size.

The interaction effects of oil-to-water ratio and Span 80 dosage on size and size distribution span are shown in Figure 7. As shown in Figure 7a, when the oil-to-water ratio was low (8), the size decreased with increasing Span 80 dosage. However, when the oil-to-water ratio exceeded 10 (e.g., 12), the size first decreased and then slowly increased with increasing Span 80 dosage. Regarding the size distribution span, as the Span 80 dosage and oil-to-water ratio increased, the size distribution span first decreased and then increased, reaching a minimum at approximately 2% Span 80 and an oil-to-water ratio of around 10 (Figure 7b).

The interaction effects of homogenization rate and Span 80 dosage on size and size distribution span are shown in Figure 8a and Figure 8b, respectively. As shown in the contour lines at the bottom of Figure 8, with increasing homogenization rate or Span 80 dosage, both size (Y1) and the size distribution span (Y2) showed a trend of first decreasing and then increasing. The effects of homogenization rate and Span 80 dosage on Y1 and Y2 are similar, but the homogenization rate and Span 80 dosage corresponding to the minimum values of Y1 and Y2 are slightly different.

Table 3 shows that the interaction effect of homogenization rate and Span 80 dosage on Y2 (size distribution span) is also significant (*p*-value < 0.0001). A higher homogenization rate leads to stronger destructive stress, promoting the formation of smaller droplets, but also increasing surface area. Thus, more Span 80 is often required to stabilize the droplets. However, due to the limited interfacial tension, once the droplets become small enough, even a sufficient amount of emulsifier is insufficient to maintain droplet stability. Furthermore, when the Span 80 concentration is too high, excess Span 80 may self-associate or aggregate at the oil–water interface, leading to aggregate formation during in situ gelation of the PHEA-HP droplets. Similar phenomena have been reported before [42]. Therefore, excessive Span 80 dosage and homogenization rate are not conducive to the formation of hydrogel beads with a narrow size distribution.

**Figure 9:**
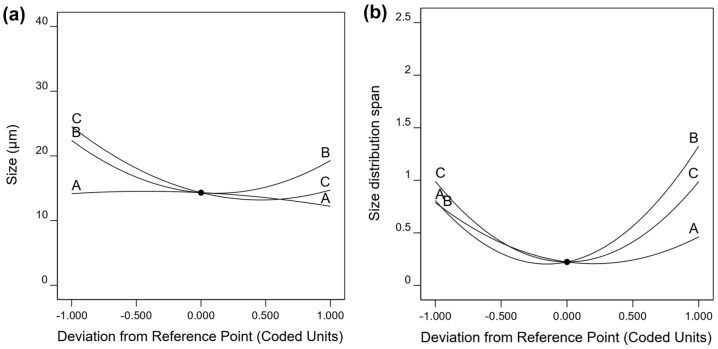
Perturbation plots for the (**a**) size and (**b**) size distribution span of PHEA-HP hydrogel beads.

In addition, Figure 9 shows the perturbation plots of the three independent variables in the model for size (Y1) and size distribution span (Y2). For size (Figure 9a), the relatively flat line for independent variable A indicates that size (Y1) is not sensitive to changes in the oil-to-water ratio. However, the curvatures of the independent variables B and C are relatively larger, indicating that size is more sensitive to the homogenization rate and Span 80 dosage than the oil-to-water ratio. The size distribution span is sensitive to all three independent variables (Figure 9b). Moreover, the curvature indicates that the size distribution span is more sensitive to the independent variables than the size itself.

It is well known that as the homogenization rate increases, the destructive stress increases, and the number of large droplets decreases drastically. However, due to the limitations of interfacial tension, the droplet size cannot be reduced further once it reaches the target size range. Therefore, given a fixed emulsifier (e.g., Span 80), increasing the homogenization rate leads to an increase in the percentage of droplets within the target size range [43]. Similarly, at a given homogenization rate, increasing the Span 80 dosage will also result in more large droplets transforming into smaller droplets within the target size range. The oil-to-water ratio is similar to that of homogenization rate and Span 80 dosage, but its impact is smaller, because the oil acts as a dispersion medium that prevents droplet coalescence. Therefore, three independent variables had a greater impact on size distribution span than on size in Figure 9.

#### 2.3.5. The Optimal Conditions

Because the model fit for Y1 (size) was inadequate, the Design-Expert optimization focused solely on minimizing Y2 (size distribution span) to find the solution. The optimal solutions determined by the BBD-RSM are as follows: (A) oil-to-water ratio = 10.3201, (B) homogenization rate = 2933.64 rpm, and (C) Span 80 dosage = 2.01597%. Under these optimal conditions, the size distribution span estimated by Design-Expert is 0.1933 with the diameter of 14.4094 μm. Considering practical operation, the final optimal conditions for preparing PHEA-HP hydrogel beads using the emulsification method are as follows: (A) oil-to-water ratio = 10.3, (B) homogenization rate = 2930 rpm, and (C) Span 80 dosage = 2.0%.

### 2.4. Characterization of Hydrogel Beads Prepared Under Optimal Conditions

**Figure 10:**
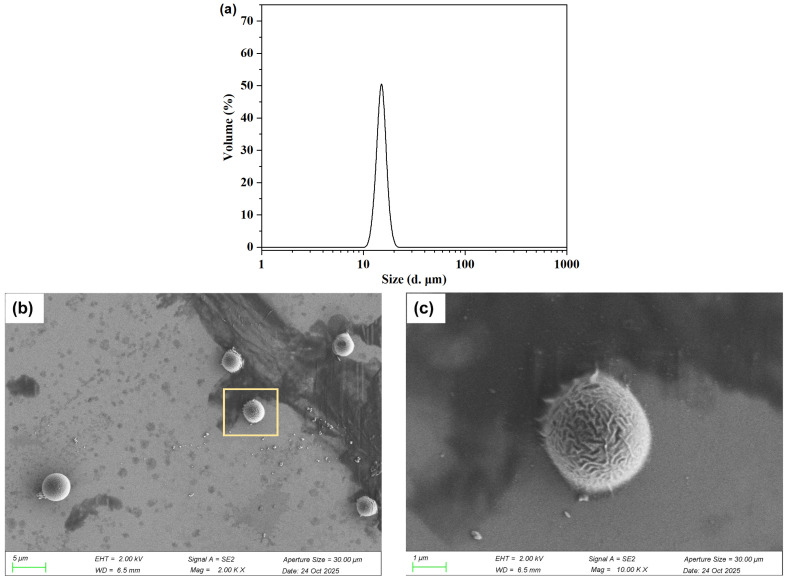
(**a**) Mastersizer result of PHEA-HP hydrogel beads prepared under the final optimal conditions, (**b**) SEM micrograph of the corresponding xerogel beads (original magnification 2000×), and (**c**) SEM micrograph of xerogel beads in the yellow rectangle in (**b**) (original magnification 10,000×).

PHEA-HP hydrogel beads were prepared under the final optimal conditions and characterized using a Mastersizer 2000 particle size analyzer (Figure 10a) and SEM (Figure 10b,c) to verify the estimated values. As shown in Figure 10a, the size distribution span of the obtained PHEA-HP hydrogel beads was 0.185 ± 0.010 with a size of 14.0 ± 0.2 μm. The difference between the actual result and the estimated value for the size distribution span (4.29%) is less than 5% according to the validation study, and the diameter is also close to the predicted value.

In addition, the shape of PHEA-HP xerogel beads was also evaluated by SEM (Figure 10b). As shown in Figure 10b,c, the obtained xerogel beads were spherical. However, the diameters of the xerogel beads observed by SEM (3–4 μm) were significantly smaller than that of the hydrogel beads measured by Mastersizer (around 14 μm). This may be attributed to the drying of the hydrogel beads prior to SEM analysis. The surface wrinkles on the xerogel beads can be clearly observed in Figure 10c. These wrinkles were formed due to the shrinkage of the hydrogel beads during drying, as previously reported [44,45]. Based on the swelling ratio of this formulation (~39.1, Figure S3), the diameter ratio of the hydrogel beads to the xerogel beads can be calculated to be approximately 3.42 (∛40.1), which is close to the size ratio measured by Mastersizer and SEM.

## 3. Conclusions

Taking advantage of the enzymatic cross-linking of PHEA-HP, PHEA-HP hydrogel beads were prepared using an emulsification method in this study. Although both size and size distribution span were evaluated as responses, analysis of variance (ANOVA) indicated that the size distribution span fit well with the quadratic model (model: *p*-value < 0.0001; lack of fit: *p*-value > 0.05 (0.6560); R^2^ and adjusted R^2^ > 0.99), and was more sensitive to the oil-to-water ratio, homogenization rate, and Span 80 dosage than size. The optimal preparation conditions were validated to be an oil-to-water ratio of 10.3, a homogenization rate of 2930 rpm, and a Span 80 dosage of 2.0%. Under these conditions, the size and size distribution span of the prepared PHEA-HP hydrogel beads were 14.0 ± 0.2 μm and 0.185 ± 0.010, respectively. This study established the model and processing parameters for preparing hydrogel beads based on an enzymatically cross-linkable poly(aspartamide) derivative.

## 4. Materials and Methods

### 4.1. Materials

Paraffin liquid (Karan ^®^) was purchased from Xi’an Tianmao Baoding Biotechnology Co., Ltd. (Xi’an, China). Hydrogen peroxide (H_2_O_2_, 30 wt.% in H_2_O) and Span 80 were purchased from Sinopharm Group (Shanghai, China). Horseradish peroxidase (HRP, RZ > 2.0, 150 units/mg) was obtained from Shanghai Macklin Biochemical Technology Co., Ltd. (Shanghai, China). Other reagents were of analytical grade and were used directly.

### 4.2. Synthesis and Characterizations of PHEA-HP

PHEA-HP was synthesized following the procedure described in our previous work [39]. The chemical structure of PHEA-HP was verified by ^1^H NMR spectrum in DMSO-d_6_, recorded on a Bruker AVANCE NEO 600 (600 MHz) NMR Spectrometer (Bruker, Bremen, Germany). The number average molecular weight (M_n_) and polydispersity index (PDI, M_w_/M_n_) of PHEA-HP were evaluated using a gel permeation chromatography (GPC) system composed of a Shimadzu LC-20AD HPLC pump (Shimadzu Corporation, Kyoto, Japan), a Shodex RI-201H refractive index detector (Showa Denko KK, Tokyo, Japan), an Agilent PLgel 5 µm MiniMIX-C guard column (50 × 4.6 mm), and an Agilent PLgel 5 µm MIXED-C column (300 × 7.5 mm) (Agilent Technologies Inc., Santa Clara, CA, USA). Calibration was performed using Polystyrene Medium EasiVial (2 mL) containing 12 polystyrene standards (PS-M, Batch Number: 0006569927, nominal molecular weight range: 162–364,000 g/mol) from Agilent Technologies (Santa Clara, CA, USA). PHEA-HP and polystyrene standards were dissolved in *N*,*N*-dimethylformamide (DMF) containing 10 mM LiBr, filtered through 0.22 μm syringe filters (nylon-6,6), and eluted by DMF with LiBr (10 mM LiBr) as a mobile phase at a flow rate of 0.5 mL/min at 35 °C. Data collection, calibration, and analysis were performed using the N2000 chromatography data system software (SP1 version) (Surwit Science Technology Co., Ltd., Hangzhou, China).

The gelation time of PHEA-HP aqueous solution was investigated by the vial-tilting method [25]. The PHEA-HP/HRP/H_2_O_2_ aqueous solution was placed in a 1.0 mL vial. If there is no flow within 20 s after inverting the vial, it is considered to have gelled. The swelling properties of the hydrogel were investigated in PBS (10 mM, pH 7.4) at room temperature. The swelling ratio was calculated using Equation (3).

(3) $$Swelling\;ratio=\frac{m_{s}-m_{d}}{m_{d}}$$

where *m_s_* is the weight of swollen hydrogel at equilibrium, and *m_d_* is the weight of dry gel.

### 4.3. Preparation of PHEA-HP Hydrogel Beads

PHEA-HP/HRP/H_2_O_2_ aqueous solution was freshly prepared as follows: (1) PHEA-HP was dissolved in deionized water first, and then divided into two equal portions. (2) HRP solution and H_2_O_2_ solution were added to each portion, respectively, to obtain PHEA-HP/HRP aqueous solution and PHEA-HP/H_2_O_2_ aqueous solution. (3) PHEA-HP/HRP and PHEA-HP/H_2_O_2_ aqueous solutions were drawn with two syringes, respectively, and the PHEA-HP/HRP/H_2_O_2_ aqueous solution was formed by direct injection through a mixing tube.

**Scheme 2:**
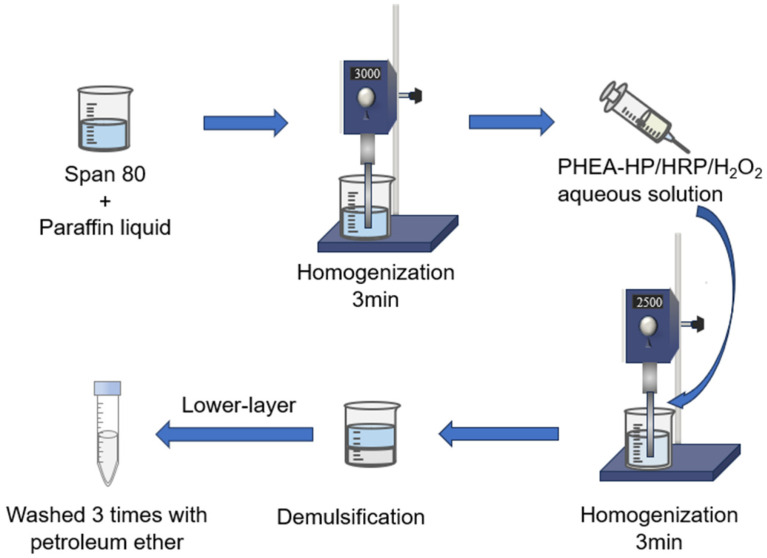
Preparation process of PHEA-HP hydrogel beads.

Liquid paraffin was used as the dispersion medium during the emulsification process because it is more stable than vegetable oils and has a relatively simple composition. The preparation process of PHEA-HP hydrogel beads by emulsification method is as follows (Scheme 2): (1) The specified amount of paraffin liquid and Span 80 were added to a beaker, stirred, and homogenized at 3000 rpm for 3 min to ensure uniform mixing; (2) Freshly prepared PHEA-HP/HRP/H_2_O_2_ aqueous solution was added to liquid paraffin containing Span 80 and immediately homogenized again for 3 min at the specified homogenization rate to form a w/o emulsion; (3) The w/o emulsion was left to stand for 10 min to allow the PHEA-HP/HRP/H_2_O_2_ aqueous droplets to cross-link in situ and complete gelation; (4) The emulsion was demulsified. The lower layer was collected and washed three times with petroleum ether to remove residual paraffin liquid and Span 80 adhering to the surface of the hydrogel beads. The collected hydrogel beads were soaked in water, with the water replaced three times to remove uncross-linked PHEA-HP. The gel yield was calculated using Equation (4).

(4) $$Gel\;yield=\frac{m_{d}}{m_{p}}$$

where *m_p_* is the feed weight of PHEA-HP. The results represent the average value of three separate experiments performed in triplicate.

### 4.4. Characterization of Hydrogel Beads

#### 4.4.1. Microscopy of Hydrogel Beads

Optical imaging of the PHEA-HP-based hydrogel beads was performed using a Soptop BH200M microscope (Sunny Optical Technology (Group) Co., Ltd., Yuyao, China) equipped with a Murzider MSD-2000 digital camera (Murzider Technology Co., Ltd., Dongguan, China) and its associated software, Murzider 4.11. The optical images were quantitatively analyzed using ImageJ 1.54.

#### 4.4.2. Size and Size Distribution

The sizes and size distributions of PHEA-HP hydrogel beads were measured by a Mastersizer 2000 particle size analyzer (Malvern Instruments, Worcestershire, UK) with a wet dispersion unit (Hydro2000 MU) at a material refractive index of 1.403, a dispersant refractive index of 1.330, and a temperature of 25 °C. The parameter “D[3,2]” (Sauter mean diameter) was used to evaluate the size (Y1) of the resulting hydrogel beads, while the parameter “span” was used to quantify the size distribution span (Y2) as it is typically used to quantify the polydispersity of the droplets [46]. The span value was calculated using Equation (5).

(5) $$span=\frac{d_{90}-d_{10}}{d_{50}}$$

where *d*_90_, *d*_50_, *d*_10_ are the diameters at 90%, 50%, and 10% cumulative volume, respectively. The results were reported as the average of triplicate measurements.

#### 4.4.3. Scanning Electron Microscopy (SEM)

Scanning electron microscopy was performed using a ZEISS GeminiSEM 300 equipped with a Schottky field emission gun (Zeiss, Oberkochen, Germany). The best results were obtained with the in-lens detector at a working distance of 6.5 mm, an aperture size of 30 µm, and an extra high tension (EHT) of 2.0 kV. The PHEA-HP hydrogel beads dispersion (0.5 wt% in water) was first dried and then coated with gold/palladium using a Quorum SC7620 Sputter Coater (Quorum Technologies Ltd., Laughton, East Sussex, UK) before the SEM measurement.

### 4.5. BBD-RSM

To investigate the effects of preparation conditions on the size and size distribution span of the hydrogel beads, the preparation conditions (oil-to-water ratio, homogenization rate, and Span 80 dosage) were optimized using a three-factor Box–Behnken design with Design-Expert software (version 13.0.1.0, Stat-Ease Inc., Minneapolis, MN, USA). The levels of the three independent variables were selected based on preliminary single-factor experiments. The Box–Behnken design generated 17 randomized experimental runs. The effects of three independent variables on hydrogel beads formation and model fitting were evaluated using analysis of variance (ANOVA) test conducted with Design-Expert software.

## Figures and Tables

**Table 1:** Independent variables, levels, and responses used in BBD-RSM.

Independent Variables	Levels		
	−1	0	1
A: Oil-to-water ratio (*v*/*v*)	8	10	12
B: Homogenization rate (rpm)	2500	3000	3500
C: Span 80 dosage (%, *w*/*v* in paraffin liquid)	1	2	3
**Responses**			
Y1: Size (μm)			
Y2: Size distribution span			

**Table 2:** Experimental results formed by BBD-RSM for the preparation of PHEA-HP hydrogel beads.

Run	A (*v*/*v*)	B (rpm)	C (%, *w*/*v*)	Y1 (μm)	Y2
1	10	3000	2	13.7 ± 0.1	0.195 ± 0.011
2	10	3000	2	13.6 ± 0.2	0.201 ± 0.004
3	10	2500	3	19.2 ± 0.2	1.290 ± 0.003
4	10	3500	3	16.1 ± 0.2	2.336 ± 0.009
5	10	3000	2	14.2 ± 0.1	0.178 ± 0.008
6	8	3000	3	14.7 ± 0.1	1.490 ± 0.005
7	10	3000	2	14.9 ± 0.0	0.235 ± 0.002
8	12	3500	2	16.0 ± 0.3	1.340 ± 0.006
9	12	3000	3	19.7 ± 0.2	1.328 ± 0.011
10	10	2500	1	37.2 ± 0.3	1.887 ± 0.008
11	10	3000	2	15.2 ± 0.1	0.309 ± 0.012
12	8	3500	2	21.3 ± 0.2	2.143 ± 0.008
13	8	2500	2	21.1 ± 0.1	1.107 ± 0.001
14	8	3000	1	23.2 ± 0.3	1.636 ± 0.005
15	12	2500	2	20.4 ± 0.2	1.287 ± 0.009
16	12	3000	1	16.4 ± 0.2	1.114 ± 0.005
17	10	3500	1	32.1 ± 0.5	1.820 ± 0.012

**Table 3:** ANOVA for the quadratic models.

	**Source**	**Sum of Squares**	**df**	**Mean Square**	**F-Value**	***p*-Value**	
Y1	Model	574.85	9	63.87	4.07	0.0389	significant
	A	7.61	1	7.61	0.4843	0.5089	
	B	19.22	1	19.22	1.22	0.3051	
	C	192.08	1	192.08	12.23	0.0100	
	AB	5.29	1	5.29	0.3369	0.5798	
	AC	34.81	1	34.81	2.22	0.1801	
	BC	1.0000	1	1.0000	0.0637	0.8080	
	A^2^	5.42	1	5.42	0.3454	0.5752	
	B^2^	178.72	1	178.72	11.38	0.0119	
	C^2^	118.94	1	118.94	7.58	0.0284	
	Residual	109.91	7	15.70			
	Lack of Fit	107.89	3	35.96	70.93	0.0006	significant
	Pure Error	2.03	4	0.5070			
	Cor Total	684.76	16				
	C.V.: 20.48%		R^2^ = 0.8395			Adjusted R^2^ = 0.6331	
	**Source**	**Sum of Squares**	**df**	**Mean Square**	**F-Value**	***p*-Value**	
Y2	Model	8.13	9	0.9028	405.51	<0.0001	significant
	A	0.2135	1	0.2135	95.91	<0.0001	
	B	0.5346	1	0.5346	240.12	<0.0001	
	C	0.0000	1	0.0000	0.0095	0.9251	
	AB	0.2416	1	0.2416	108.51	<0.0001	
	AC	0.0324	1	0.0324	14.55	0.0066	
	BC	0.3097	1	0.3097	139.11	<0.0001	
	A^2^	0.6811	1	0.6811	305.94	<0.0001	
	B^2^	3.00	1	3.00	1345.47	<0.0001	
	C^2^	2.47	1	2.47	1110.30	<0.0001	
	Residual	0.0156	7	0.0022			
	Lack of Fit	0.0048	3	0.0016	0.5851	0.6560	not significant
	Pure Error	0.0108	4	0.0027			
	Cor Total	8.14	16				
	C.V.: 4.03%		R^2^ = 0.9981			Adjusted R^2^ = 0.9956	

## Data Availability

All data is contained within this article or the Supplementary Materials.

## Supplementary Materials

The following supporting information can be downloaded at: https://www.mdpi.com/article/10.3390/gels12030230/s1, Figure S1: ^1^H NMR spectrum of PHEA-HP in DMSO-d_6_; Figure S2: Gelation time of PHEA-HP aqueous solution as a function of the concentrations of HRP and H_2_O_2_ (PHEA-HP concentration: 6.0 wt.%); Figure S3: Swelling kinetics of a PHEA-HP hydrogel in PBS. Gel formulation: 6.0 wt.% PHEA-HP, 0.5 units/mL HRP, 10 mM H_2_O_2_; Figure S4: The size distributions of PHEA-HP hydrogel beads prepared at varying oil-to-water ratios: (a) 6, (b) 8, (c) 10, and (d) 12. (Homogenization rate: 3000 rpm; Span 80: 2% *w*/*v* in paraffin liquid); Figure S5: The size distributions of PHEA-HP hydrogel beads prepared at varying homogenization rates: (a) 2000 rpm, (b) 2500 rpm, and (c) 3000 rpm. (Oil-to-water ratio: 10; Span 80: 2% *w*/*v* in paraffin liquid); Figure S6: The size distributions of PHEA-HP hydrogel beads prepared at varying Span 80 dosages (*w*/*v* in paraffin liquid): (a) 1%, (b) 2%, and (c) 3% (oil-to-water ratio: 10; homogenization rate: 3000 rpm); Figure S7: The representative size distributions of PHEA-HP hydrogel beads: (a) Run 2, (b) Run 3, (c) Run 12, and (d) Run 14.
