# Supplementary material for: Optimization of Emulsification Parameters for Preparing Hydrogel Beads Based on an Enzymatically Cross-Linkable Poly(aspartamide) Derivative
Source: Gels. 2026 Mar 11;12(3):230. doi: 10.3390/gels12030230 (PMC13025965; doi:10.3390/gels12030230)
Supplement: Supplementary file 1 [file gels-12-00230-s001.zip › gels-4181426-supplementary.pdf]

Supplementary Materials

Optimization of Emulsification Parameters for Preparing Hydrogel Beads based on an Enzymatically Cross-linkable Poly(aspartamide) Derivative

Danqing Liu [1] and Guangyan Zhang [1,2,*]

[1] School of Materials and Chemical Engineering, Hubei University of Technology, Wuhan 430068, China;
[2] Hubei Provincial Key Laboratory of Green Materials for Light Industry, Hubei University of Technology, Wuhan 430068, China
* Correspondence: gyzhang@hbut.edu.cn (G.Z.)

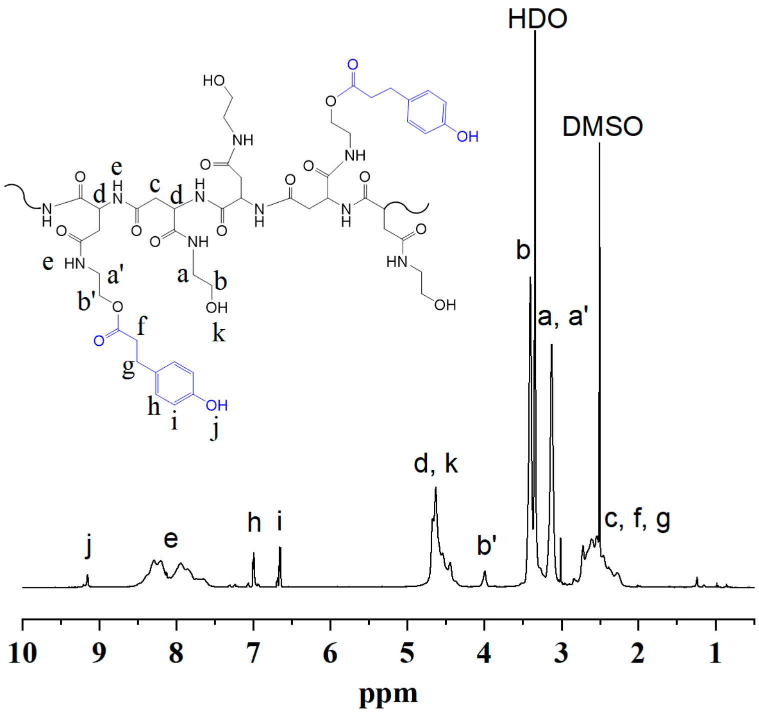

Figure S1. ^{1}H NMR spectrum of PHEA-HP in DMSO-d_6.

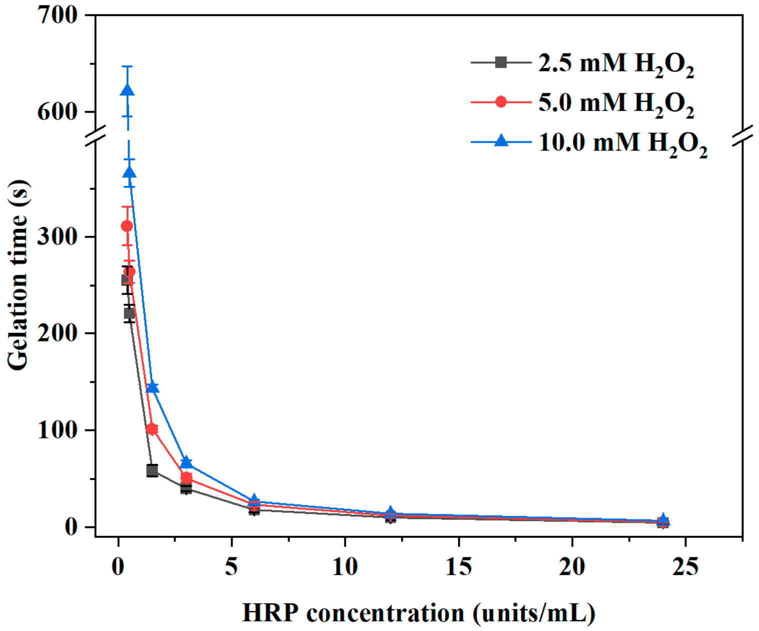

Figure S2. Gelation time of PHEA-HP aqueous solution as a function of the concentrations of HRP and H_2O_2 (PHEA-HP concentration: 6.0 wt.%).

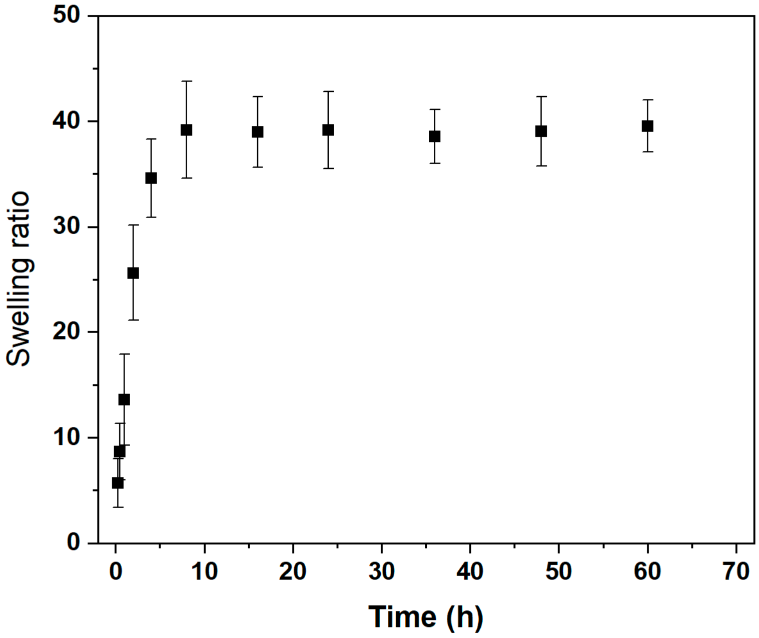

Figure S3. Swelling kinetics of a PHEA-HP hydrogel in PBS. Gel formulation: 6.0 wt.% PHEA-HP, 0.5 units/mL HRP, 10 mM H_2O_2.

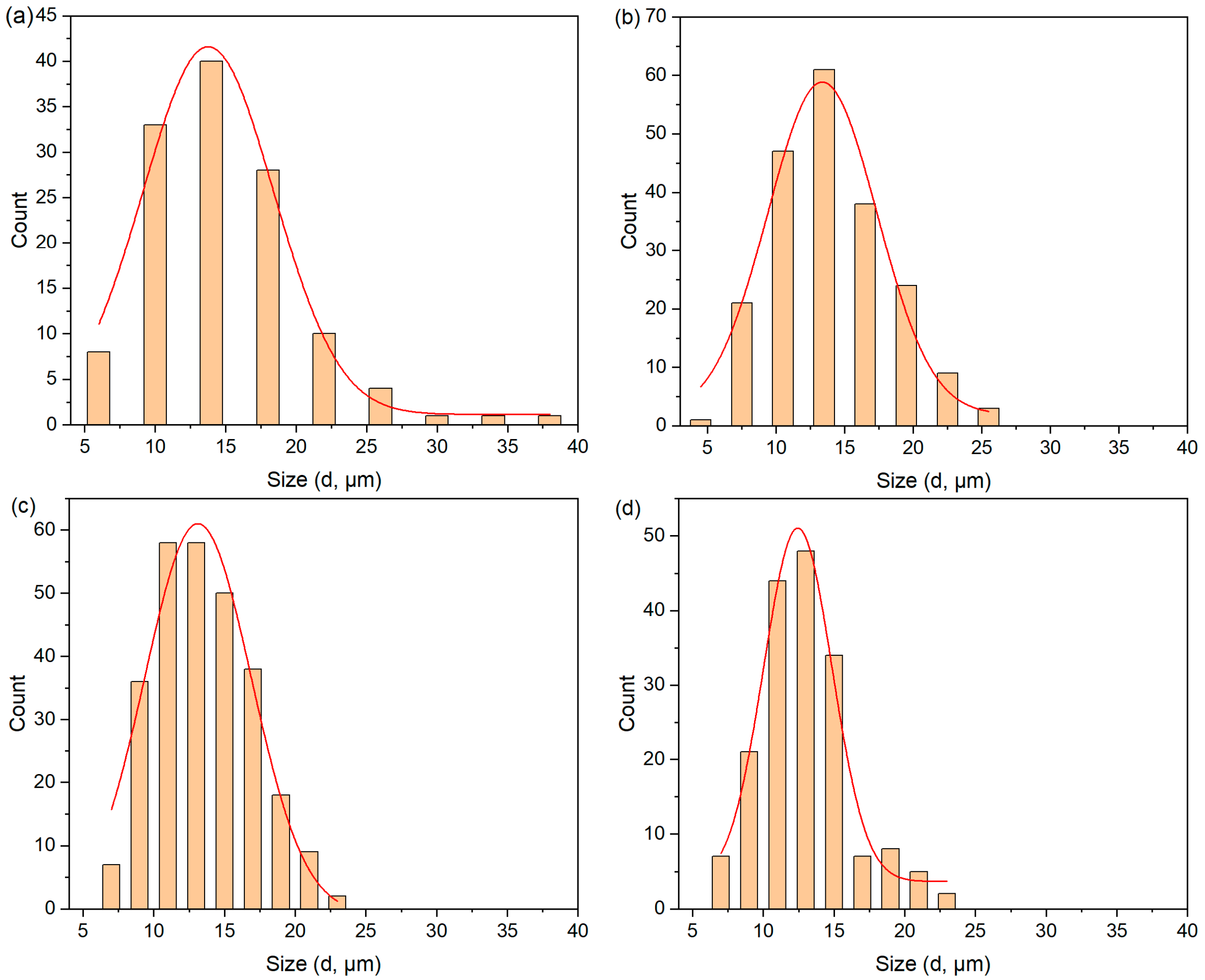

Figure S4. The size distributions of PHEA-HP hydrogel beads prepared at varying oil-to-water ratios: (a) 6, (b) 8, (c) 10, and (d) 12. (Homogenization rate: 3000 rpm; Span 80: 2% w/v in paraffin liquid).

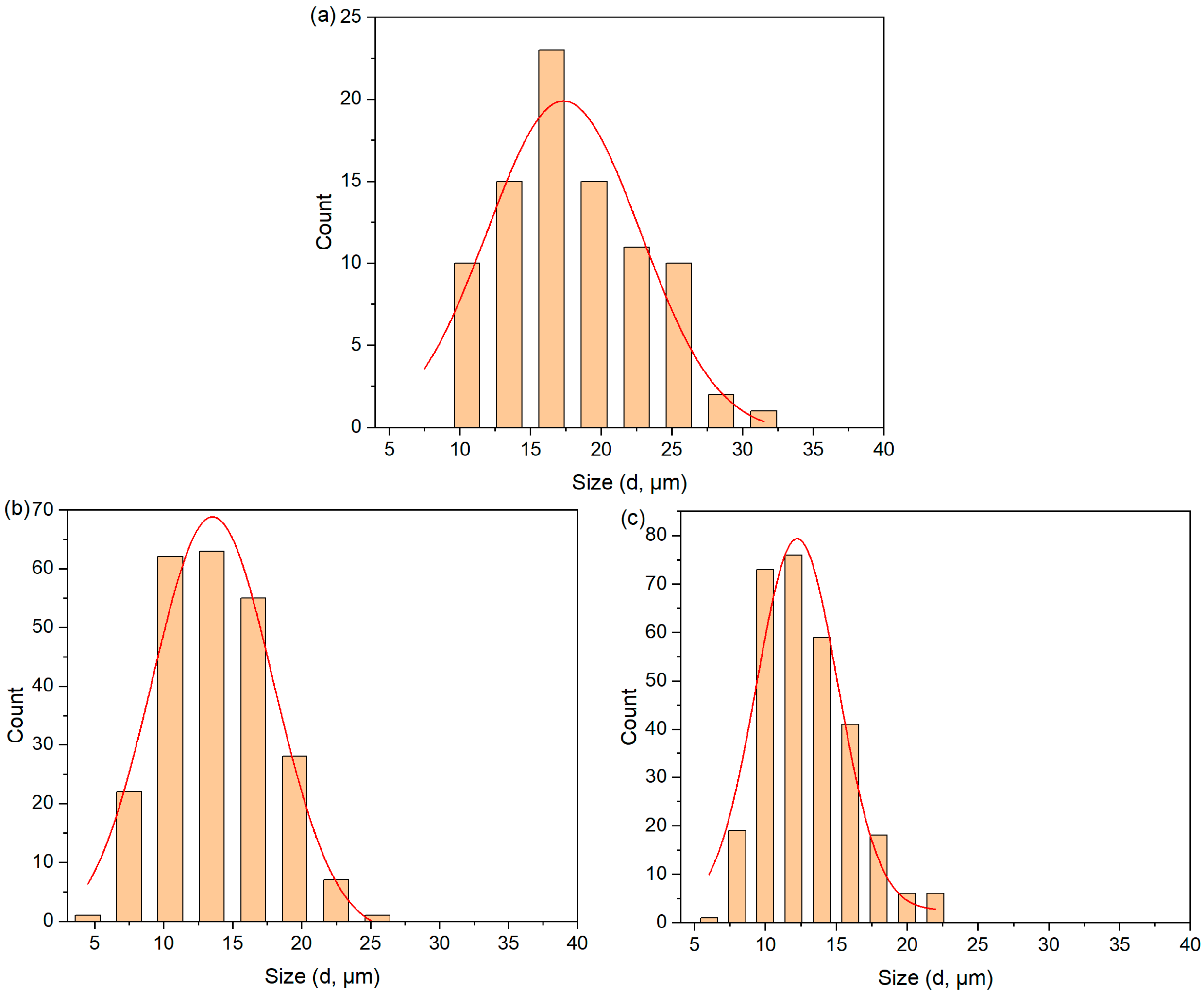

Figure S5. The size distributions of PHEA-HP hydrogel beads prepared at varying homogenization rates: (a) 2000 rpm, (b) 2500 rpm, and (c) 3000 rpm. (Oil-to-water ratio: 10; Span 80: 2% w/v in paraffin liquid).

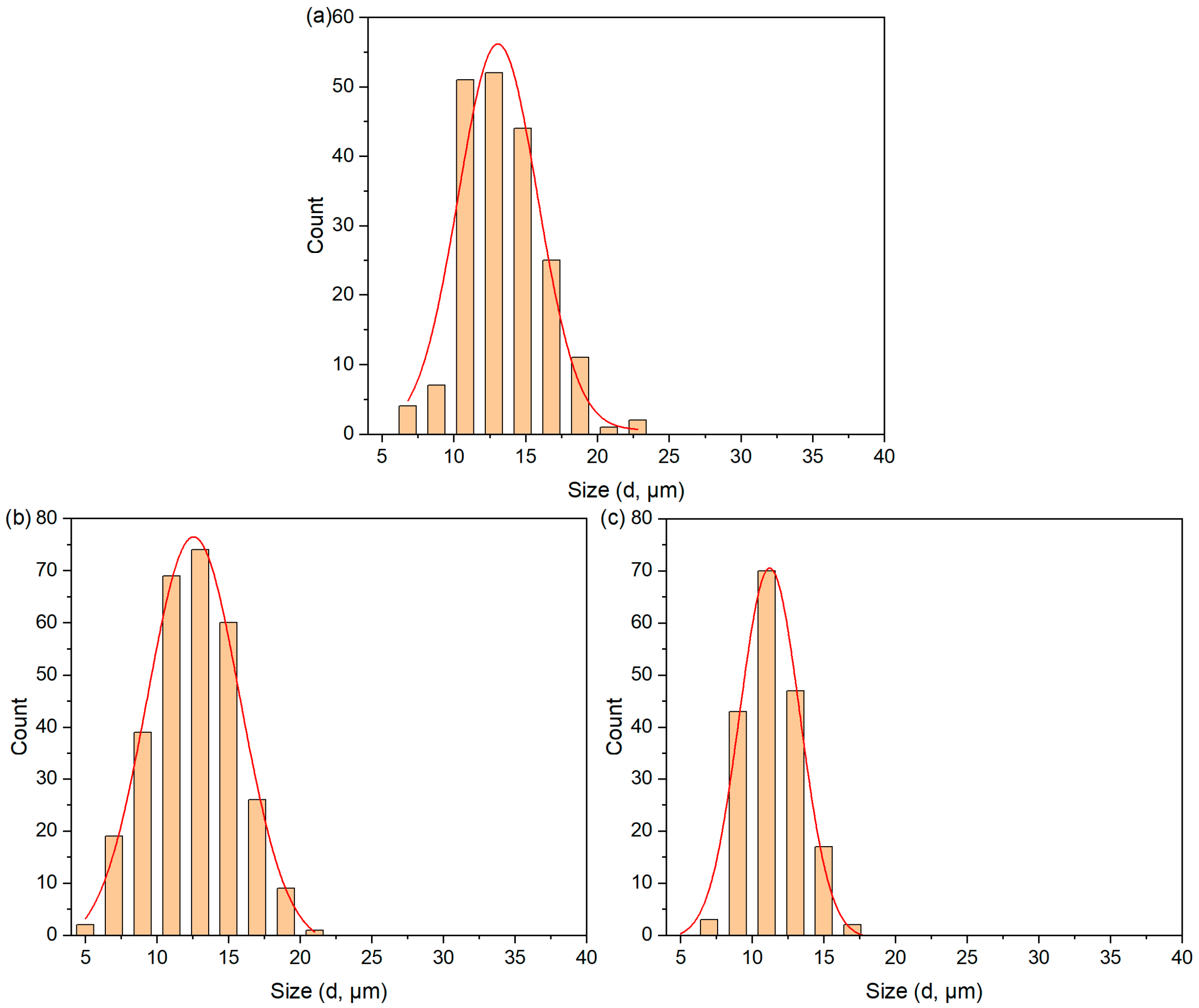

Figure S6. The size distributions of PHEA-HP hydrogel beads prepared at varying Span 80 dosages (w/v in paraffin liquid): (a) 1%, (b) 2%, and (c) 3%. (Oil-to-water ratio: 10; homogenization rate: 3000 rpm).

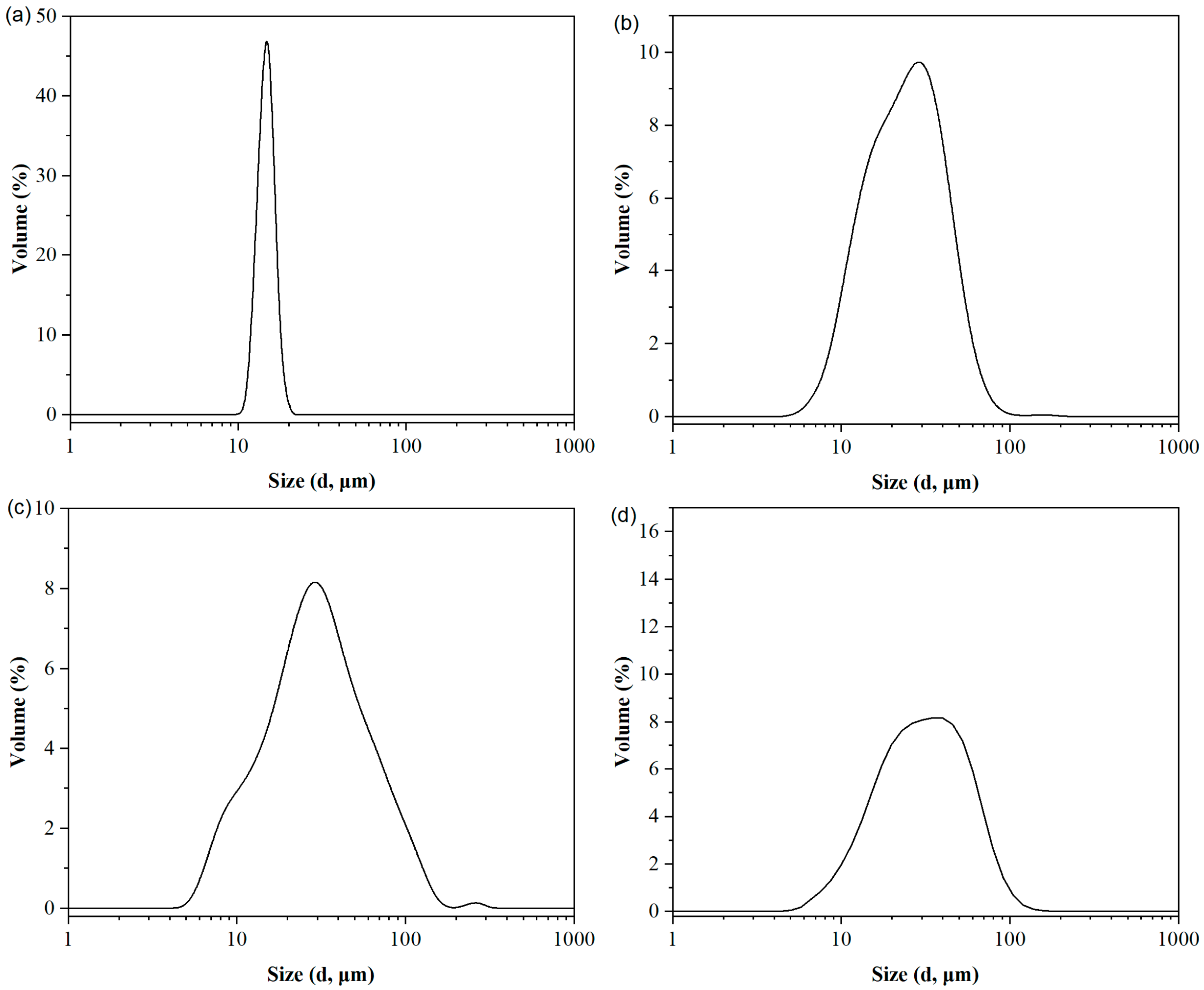

Figure S7. The representative size distributions of PHEA-HP hydrogel beads: (a) Run 2, (b) Run 3, (c) Run 12, and (d) Run 14.
